# Trends in mortality rates associated with multiple myeloma in the United States, 1999–2023

**DOI:** 10.3389/fonc.2025.1735565

**Published:** 2025-12-12

**Authors:** Ye Jiang, Guanghua Liang, Yongping Cao

**Affiliations:** Department of Orthopedics, Peking University First Hospital, Beijing, China

**Keywords:** multiple myeloma, mortality trends, CDC WONDER, age-adjusted mortality rate, USA

## Abstract

**Background:**

Despite substantial therapeutic advances, multiple myeloma (MM) continues to be an important contributor to cancer-related mortality in the United States. Understanding long-term mortality patterns is essential for guiding equitable clinical and public health strategies.

**Methods:**

We used CDC WONDER mortality data from 1999–2023 to evaluate MM-related deaths among U.S. adults aged ≥25 years. Age-adjusted mortality rates (AAMRs) were calculated using direct standardization to the 2000 U.S. population. Temporal trends were examined using Joinpoint regression to estimate annual percent changes (APCs) and identify significant shifts over time. Analyses were stratified by age, sex, race/ethnicity, state, and urbanization level.

**Results:**

Nationally, MM mortality declined over the study period. Most age groups younger than 85 years showed statistically significant decreases in AAMR, whereas adults aged ≥85 years experienced a modest increase (APC = +0.67; 95% CI, 0.48–0.86). Males consistently exhibited higher AAMRs than females, although both showed declining trends. Substantial and persistent disparities were observed across racial and ethnic groups, as well as geographic and urbanization categories. Notable state-level heterogeneity was evident, with some states—including New York, Ohio, and Kansas—showing accelerated recent declines in mortality.

**Conclusion:**

From 1999 to 2023, MM-related mortality in the U.S. declined overall, but substantial and persistent disparities remained by age (notably a rising burden among the oldest adults), race/ethnicity, sex, geography, and urbanization. These findings underscore the need for targeted research that links diagnosis, treatment uptake, and outcomes, and for policies to ensure equitable access to proven MM therapies so that survival gains are distributed more uniformly across all population groups.

## 1 Introduction

Multiple myeloma (MM) is a clonal plasma cell proliferative disorder originating from plasma cells. It is characterized by abnormal proliferation of plasma cells in the bone marrow and the production of monoclonal immunoglobulins, which can lead to end-organ damage such as bone pain, anemia, renal dysfunction, and hypercalcemia. MM accounts for approximately 10% of all hematologic malignancies. Despite substantial advances in diagnostic techniques and therapeutic strategies, including the development of novel agents (1, 2), MM remains an active and evolving field within clinical oncology and is still regarded as an incurable malignancy (3–7).

In the United States, MM represents approximately 2% of newly diagnosed cancers and contributes to a similar proportion of cancer-related deaths. Compared with global patterns, the incidence of MM in the U.S. has shown a gradual upward trend over the past several decades, whereas overall mortality has declined modestly (3, 8–10)737. For instance, a study based on SEER and CDC WONDER data from 1999 to 2020 demonstrated that while MM incidence steadily increased, age-adjusted mortality rates (AAMRs) consistently declined, with significant disparities observed among racial and ethnic groups (11). Additionally, an investigation into MM mortality inequities across the U.S. revealed that the rate of AAMR decline varied among population subgroups, further exacerbating public health challenges (12).

Although previous studies have focused on trends in MM incidence and survival improvement, comprehensive analyses of mortality trends and influencing factors among the general U.S. population between 1999 and 2023 remain limited. In particular, with the broader implementation of treatment modalities such as autologous stem cell transplantation, targeted therapies, and immunotherapies, it remains unclear whether emerging mortality patterns exhibit heterogeneity across sex, race/ethnicity, age groups, geographic regions, or levels of urbanization. Existing evidence suggests that different subgroups (e.g., males vs. females, Black vs. White individuals) display variations in MM incidence, prognosis, and mortality risk (3). For example, studies have shown that although African Americans are diagnosed with MM at more than twice the rate of non-Hispanic Whites, the mortality gap between these groups persists, underscoring ongoing health inequities (13, 14). Moreover, differences in treatment accessibility, healthcare resource distribution, socioeconomic status, and demographic changes may play crucial roles in shaping these divergent trends (15–17).

To better understand mortality trends and their determinants in MM, epidemiological analyses are essential. The Centers for Disease Control and Prevention (CDC) Wide-ranging Online Data for Epidemiologic Research (WONDER) database serves as a vital national public health resource, providing U.S. mortality data since 1999. This database offers valuable insights for examining temporal trends and demographic disparities in MM-related deaths (18, 19). Therefore, this study systematically analyzes underlying-cause-of-death records for MM from 1999 to 2023 using the CDC WONDER database. Specifically, we aim to evaluate temporal variations in age-adjusted mortality rates (AAMRs) overall and by sex, race/ethnicity, age group, geographic region, state, and level of urbanization; quantify percentage changes between 1999 and 2023; estimate annual and average annual percent changes (APC/AAPC); and explore potential determinants and regional heterogeneity underlying these trends. By identifying persistent health disparities among population subgroups, this study seeks to provide an evidence base for public health policy formulation, equitable resource allocation, and the prioritization of interventions—ultimately contributing to improved outcomes and survival among patients with multiple myeloma.

## 2 Materials and methods

### 2.1 Study sample and data source

We retrieved mortality data for individuals aged 25 years and older whose underlying cause of death was attributed to multiple myeloma (MM) from the CDC WONDER database for the years 1999–2023. The CDC Wide-ranging Online Data for Epidemiologic Research (WONDER) system, developed by the U.S. Centers for Disease Control and Prevention (CDC), provides access to publicly available, processed mortality data derived from death certificates within the National Vital Statistics System. It serves as a comprehensive source of public health indicators and demographic information, including underlying cause of death, age, sex, race/ethnicity, and place of death. Because all data are de-identified and aggregated for public use, this study did not require informed consent or institutional review board (IRB) approval.

Mortality rate—defined as the frequency of deaths within a specified population during a given time period—is typically expressed per 100,000 population. To identify deaths specifically attributable to multiple myeloma, we used the “underlying cause of death” records available in the CDC WONDER database. Cases were identified according to the International Classification of Diseases, 10th Revision (ICD-10) codes in the C90 series (particularly C90.0) corresponding to MM as the underlying cause of death. Extracted variables included basic demographic information (e.g., state or county of residence, sex, age, race/ethnicity) and year of death. As all data were anonymized and publicly accessible in aggregated form, no informed consent or ethical approval was required for this analysis.

### 2.2 Data analysis

Mortality data extracted from the CDC WONDER database were aggregated as death counts and mortality rates per 100,000 population, stratified by sex, age group, and race/ethnicity. Racial and ethnic categories followed the U.S. Census conventions and included non-Hispanic White, non-Hispanic Black, Hispanic, non-Hispanic Asian/Pacific Islander, and other/unknown groups. Geographic stratifications encompassed state-level data, the four major U.S. Census regions (Northeast, Midwest, South, and West), nine Census divisions, and ten U.S. Department of Health and Human Services (HHS) regions. Urbanization levels were classified according to the metropolitan/nonmetropolitan scheme commonly used in National Vital Statistics Reports, comprising large central metro, large fringe metro, medium metro, small metro, micropolitan/small town, and noncore/nonmetropolitan categories. The study period covered data from 1999 through 2023.

Crude mortality rates were calculated as the number of deaths divided by the corresponding population and expressed per 100,000 individuals. Age-adjusted mortality rates (AAMRs) were computed by direct standardization to the 2000 U.S. standard population to facilitate comparisons across demographic subgroups and time periods. Percent change between 1999 and 2023 was calculated as [(rate_2023 − rate_1999)/rate_1999] × 100%.

Temporal trend analyses were conducted using the Joinpoint Regression Program (National Cancer Institute, USA) to identify statistically significant inflection points and to estimate annual percent change (APC) for each segment, as well as the average annual percent change (AAPC) for specified intervals. Both APC and AAPC were reported with 95% confidence intervals (CIs), and statistical significance was determined via the program’s built-in permutation test at a two-sided significance level of p < 0.05. For segmented trends, time intervals were defined according to the breakpoints identified by the Joinpoint model.

Cells with suppressed or small counts (due to CDC WONDER’s confidentiality thresholds) were treated as missing (NA) and excluded from analyses requiring reliable estimates; no imputation was performed. All analyses were based solely on available (non-suppressed) data. The number of included deaths and sample sizes for each stratum are detailed in the tables and figure legends.

To visualize spatial and temporal variation, state- and region-level choropleth maps and time-series plots were generated. Figures and summary tables were produced using R software (and associated visualization packages) and were cross-validated using spreadsheet software to ensure consistency and accuracy.

## 3 Results

### 3.1 Geographic and temporal trends in U.S. multiple myeloma mortality

Based on the CDC WONDER database, this study analyzed trends in multiple myeloma (MM)–related mortality in the United States from 1999 to 2023. As summarized in **Table 1**, a total of 288,946 MM-related deaths were recorded during this period. The number of deaths increased from 10,565 in 1999 to 11,766 in 2023, representing an overall increase of 11.37%. However, the age-adjusted mortality rate (AAMR) decreased significantly from 5.97 (95% CI: 5.86–6.09) per 100,000 population in 1999 to 4.20 (95% CI: 4.12–4.28) in 2023, with an average annual percent change (AAPC) of –1.54% (95% CI: –2.08 to –1.01).

**Table 1:** Trends in multiple myeloma–related mortality in the United States, 1999–2023.

Characteristic		Deaths 1999	Deaths 2023	Deaths 1999-2023	Change(%)	AAMR 1999	AAMR 2023	AAPC (95% CI)
Gender	Both	10565	11766	288946	11.37	5.97 (5.86 to 6.09)	4.20 (4.12 to 4.28)	-1.54 (-2.08 to -1.01)*
	Female	5227	5366	133545	2.66	5.07 (4.93 to 5.20)	3.42 (3.33 to 3.52)	-1.66 (-2.23 to -1.08)*
	Male	5338	6400	155401	19.90	7.31 (7.12 to 7.51)	5.17 (5.04 to 5.30)	-1.47 (-1.86 to -1.07)*
Census region	Northeast	2097	1975	52880	-5.82	5.65 (5.41 to 5.89)	3.80 (3.63 to 3.97)	-1.72 (-2.18 to -1.26)*
	Midwest	2518	2586	67394	2.70	6.00 (5.76 to 6.23)	4.40 (4.23 to 4.57)	-1.35 (-2.01 to -0.69)*
	South	3935	4772	110867	21.27	6.25 (6.06 to 6.45)	4.48 (4.35 to 4.61)	-1.31 (-1.96 to -0.65)*
	West	2015	2433	57805	20.74	5.71 (5.46 to 5.96)	3.84 (3.69 to 4.00)	-1.26 (-1.46 to -1.06)*
Race	Hispanic	461	1016	18681	120.39	4.91 (4.44 to 5.38)	3.57 (3.35 to 3.80)	-1.29 (-1.55 to -1.03)*
	NH Black	1763	2160	50718	22.52	11.65 (11.10 to 12.20)	7.97 (7.62 to 8.31)	-1.42 (-2.18 to -0.65)*
	NH White	8133	8162	212000	0.36	5.49 (5.37 to 5.61)	3.94 (3.86 to 4.03)	-1.39 (-1.93 to -0.85)*
	NH Other	192	362	6913	88.54	3.65 (3.11 to 4.19)	2.18 (1.96 to 2.41)	-1.33 (-1.71 to -0.94)*
Urbanization level	Large Central Metro	3073	3389	69658	10.28	6.19 (5.97 to 6.40)	4.60 (4.44 to 4.75)	-1.33 (-1.78 to -0.87)*
	Large Fringe Metro	2310	3052	59013	32.12	5.80 (5.57 to 6.04)	4.53 (4.36 to 4.69)	-1.18 (-1.37 to -1.00)*
	Medium Metro	2168	2773	53539	27.91	5.89 (5.64 to 6.13)	4.76 (4.58 to 4.94)	-1.16 (-1.89 to -0.43)*
	Small Metro	1008	1270	25311	25.99	5.82 (5.46 to 6.18)	4.79 (4.53 to 5.06)	-1.22 (-2.40 to -0.02)*
	Micropolitan (Nonmetro)	1077	1204	25167	11.79	5.88 (5.53 to 6.24)	4.79 (4.51 to 5.06)	-1.09 (-1.30 to -0.88)*
	NonCore (Nonmetro)	929	900	19729	-3.12	6.18 (5.78 to 6.58)	4.60 (4.30 to 4.91)	-1.14 (-1.44 to -0.84)*
State	Alabama	171	197	5216	15.20	5.83 (4.95 to 6.70)	4.46 (3.83 to 5.09)	-1.58 (-1.98 to -1.18)*
	Alaska	15	20	NA	33.33	NA (3.94 to 12.64)	3.82 (2.26 to 6.04)	NA
	Arizona	170	225	5388	32.35	5.21 (4.42 to 6.00)	3.32 (2.88 to 3.76)	-1.11 (-1.58 to -0.65)*
	Arkansas	119	112	2915	-5.88	6.37 (5.22 to 7.52)	4.41 (3.58 to 5.24)	-1.42 (-1.83 to -1.01)*
	California	1051	1212	28771	15.32	5.72 (5.37 to 6.07)	3.93 (3.71 to 4.15)	-1.20 (-1.39 to -1.00)*
	Colorado	114	168	3887	47.37	5.24 (4.28 to 6.21)	3.90 (3.30 to 4.49)	-1.10 (-1.70 to -0.50)*
	Connecticut	137	112	3311	-18.25	5.72 (4.76 to 6.67)	3.35 (2.72 to 3.98)	-1.63 (-2.12 to -1.13)*
	Delaware	22	43	985	95.45	4.24 (2.66 to 6.42)	4.40 (3.15 to 5.96)	-0.23 (-1.02 to 0.56)
	District of Columbia	35	20	735	-42.86	9.76 (6.80 to 13.57)	4.43 (2.70 to 6.84)	NA
	Florida	711	881	20274	23.91	5.34 (4.94 to 5.74)	3.75 (3.49 to 4.00)	-0.83 (-1.14 to -0.53)*
	Georgia	267	371	8373	38.95	6.39 (5.61 to 7.16)	4.67 (4.18 to 5.15)	-1.16 (-1.56 to -0.76)*
	Hawaii	38	45	1002	18.42	4.73 (3.35 to 6.49)	3.17 (2.30 to 4.27)	-0.86 (-1.67 to -0.04)*
	Idaho	48	73	1407	52.08	6.45 (4.76 to 8.56)	4.66 (3.64 to 5.88)	-0.84 (-1.68 to 0.01)
	Illinois	487	407	11712	-16.43	6.36 (5.79 to 6.92)	3.96 (3.57 to 4.35)	-1.60 (-1.81 to -1.39)*
	Indiana	225	251	6234	11.56	5.85 (5.09 to 6.62)	4.62 (4.04 to 5.20)	-1.28 (-1.71 to -0.84)*
	Iowa	119	127	3300	6.72	5.37 (4.40 to 6.34)	4.37 (3.60 to 5.14)	-0.78 (-1.28 to -0.27)*
	Kansas	97	111	2856	14.43	5.41 (4.38 to 6.60)	4.64 (3.77 to 5.52)	-1.45 (-2.35 to -0.53)*
	Kentucky	170	163	4243	-4.12	6.61 (5.61 to 7.60)	4.42 (3.73 to 5.11)	-1.20 (-1.62 to -0.77)*
	Louisiana	177	189	4577	6.78	6.71 (5.72 to 7.70)	5.13 (4.38 to 5.87)	-1.12 (-1.52 to -0.72)*
	Maine	64	59	1453	-7.81	6.92 (5.33 to 8.84)	3.85 (2.92 to 4.98)	-1.70 (-2.41 to -0.98)*
	Maryland	226	261	6059	15.49	7.22 (6.27 to 8.16)	5.08 (4.46 to 5.71)	-0.87 (-1.27 to -0.47)*
	Massachusetts	239	259	6241	8.37	5.51 (4.81 to 6.21)	4.23 (3.71 to 4.75)	-1.40 (-1.76 to -1.04)*
	Michigan	390	455	10727	16.67	6.29 (5.66 to 6.91)	5.12 (4.64 to 5.60)	-0.98 (-1.31 to -0.66)*
	Minnesota	179	215	5178	20.11	5.78 (4.93 to 6.63)	4.42 (3.82 to 5.01)	-1.12 (-1.51 to -0.72)*
	Mississippi	101	144	3086	42.57	5.82 (4.68 to 6.95)	5.88 (4.91 to 6.86)	0.08 (-0.39 to 0.55)
	Missouri	218	207	6014	-5.05	5.73 (4.96 to 6.49)	3.87 (3.33 to 4.40)	-1.34 (-1.77 to -0.91)*
	Montana	34	55	1062	61.76	5.53 (3.83 to 7.73)	5.45 (4.08 to 7.12)	-0.90 (-1.72 to -0.08)*
	Nebraska	62	83	1741	33.87	5.37 (4.12 to 6.89)	5.28 (4.19 to 6.56)	-0.76 (-1.23 to -0.30)*
	Nevada	43	79	1926	83.72	4.06 (2.91 to 5.50)	3.00 (2.36 to 3.75)	-1.39 (-2.26 to -0.50)*
	New Hampshire	33	41	1247	24.24	4.35 (3.00 to 6.12)	3.10 (2.20 to 4.24)	-1.29 (-2.29 to -0.27)*
	New Jersey	317	298	8191	-5.99	5.63 (5.01 to 6.25)	3.76 (3.33 to 4.19)	-1.76 (-2.19 to -1.33)*
	New Mexico	58	65	1722	12.07	5.44 (4.13 to 7.03)	3.41 (2.62 to 4.37)	-1.45 (-2.19 to -0.70)*
	New York	689	592	16950	-14.08	5.57 (5.15 to 5.99)	3.39 (3.12 to 3.67)	-1.90 (-2.40 to -1.40)*
	North Carolina	338	380	9824	12.43	6.89 (6.15 to 7.62)	4.34 (3.90 to 4.78)	-1.42 (-1.85 to -0.99)*
	North Dakota	27	26	728	-3.70	5.69 (3.71 to 8.33)	4.02 (2.60 to 5.94)	NA
	Ohio	472	454	12325	-3.81	6.21 (5.65 to 6.77)	4.41 (4.00 to 4.83)	-1.35 (-1.86 to -0.84)*
	Oklahoma	85	179	3568	110.59	3.71 (2.96 to 4.58)	5.76 (4.90 to 6.61)	0.18 (-0.48 to 0.84)
	Oregon	148	161	3967	8.78	6.56 (5.50 to 7.62)	4.20 (3.54 to 4.85)	-1.45 (-1.90 to -1.01)*
	Pennsylvania	556	538	13889	-3.24	5.83 (5.35 to 6.32)	4.36 (3.98 to 4.73)	-1.03 (-1.31 to -0.74)*
	Rhode Island	38	44	983	15.79	5.08 (3.60 to 6.98)	4.17 (3.00 to 5.63)	-0.90 (-1.56 to -0.24)*
	South Carolina	177	249	5369	40.68	7.22 (6.15 to 8.29)	5.34 (4.66 to 6.02)	-1.22 (-1.78 to -0.65)*
	South Dakota	27	32	897	18.52	5.01 (3.30 to 7.29)	4.25 (2.87 to 6.07)	-1.20 (-2.28 to -0.11)*
	Tennessee	253	281	6737	11.07	7.03 (6.16 to 7.90)	4.81 (4.24 to 5.38)	-1.44 (-1.71 to -1.16)*
	Texas	659	878	18983	33.23	6.20 (5.72 to 6.67)	4.40 (4.10 to 4.69)	-1.27 (-1.61 to -0.92)*
	Utah	61	71	1797	16.39	6.22 (4.76 to 7.99)	3.73 (2.90 to 4.72)	-1.53 (-2.26 to -0.80)*
	Vermont	24	32	615	33.33	6.03 (3.87 to 8.98)	4.94 (3.36 to 7.01)	NA
	Virginia	332	351	2073	5.72	8.10 (7.22 to 8.97)	4.92 (4.40 to 5.44)	-1.39 (-1.75 to -1.03)*
	Washington	226	238	6021	5.31	6.56 (5.70 to 7.41)	3.87 (3.37 to 4.37)	-1.62 (-2.04 to -1.19)*
	West Virginia	92	73	2073	-20.65	6.65 (5.36 to 8.15)	4.02 (3.14 to 5.07)	-1.03 (-1.59 to -0.47)*
	Wisconsin	215	218	5682	1.40	6.04 (5.24 to 6.85)	4.13 (3.58 to 4.69)	-1.05 (-1.71 to -0.38)*
	Wyoming	NA	21	NA	NA	NA (NA to NA)	4.04 (2.47 to 6.24)	NA
Age	25–34 years	21	NA	NA	NA	0.05 (0.03 to 0.08)	NA (NA to NA)	NA
	35–44 years	155	70	2822	-54.84	0.34 (0.29 to 0.40)	0.16 (0.12 to 0.20)	-2.70 (-3.38 to -2.01)*
	45–54 years	649	399	15170	-38.52	1.77 (1.64 to 1.91)	0.99 (0.89 to 1.08)	-2.57 (-3.44 to -1.69)*
	55–64 years	1639	1393	43685	-15.01	6.89 (6.56 to 7.23)	3.33 (3.15 to 3.50)	-2.68 (-3.36 to -2.00)*
	65–74 years	3075	3194	79455	3.87	16.69 (16.10 to 17.28)	9.21 (8.89 to 9.53)	-2.50 (-2.89 to -2.10)*
	75–84 years	3619	4201	96624	16.08	29.60 (28.64 to 30.57)	22.87 (22.18 to 23.56)	-1.19 (-1.77 to -0.61)*
	85+ years	1407	2500	50948	77.68	33.87 (32.10 to 35.64)	40.36 (38.77 to 41.94)	0.67 (0.48 to 0.86)*

AAMR, age-adjusted mortality rate; AAPC, average annual percent change; CI, confidence interval.

*Indicates statistically significant change (p < 0.05). Data are for U.S. adults aged ≥25 years. Counts suppressed in CDC WONDER or cells with small counts were treated as missing and excluded from rate calculations. In the Urbanization section, the 2023 data used the 2020 data as a substitute, and the AAPC was calculated for the period from 1999 to 2020. In the age group section, AAMR used the crude mortality rate as a substitute, and the AAPC calculated the crude mortality rate.

Stratified analyses revealed consistent declines in AAMR across genders, census regions, racial/ethnic groups, and urbanization levels, though disparities persisted. Males and Non-Hispanic Black individuals experienced the highest AAMRs throughout the period. Geographically, the Northeast reported a decline in death counts (–5.82%), while the South and West saw increases exceeding 20%. By age group, notable mortality reductions were observed among individuals aged 35–44, 45–54, and 55–64 years, whereas the oldest group (85+ years) experienced a substantial increase in both death counts and AAMR (**Table 1**, **Supplementary Table 1**).

**Figure 1:**
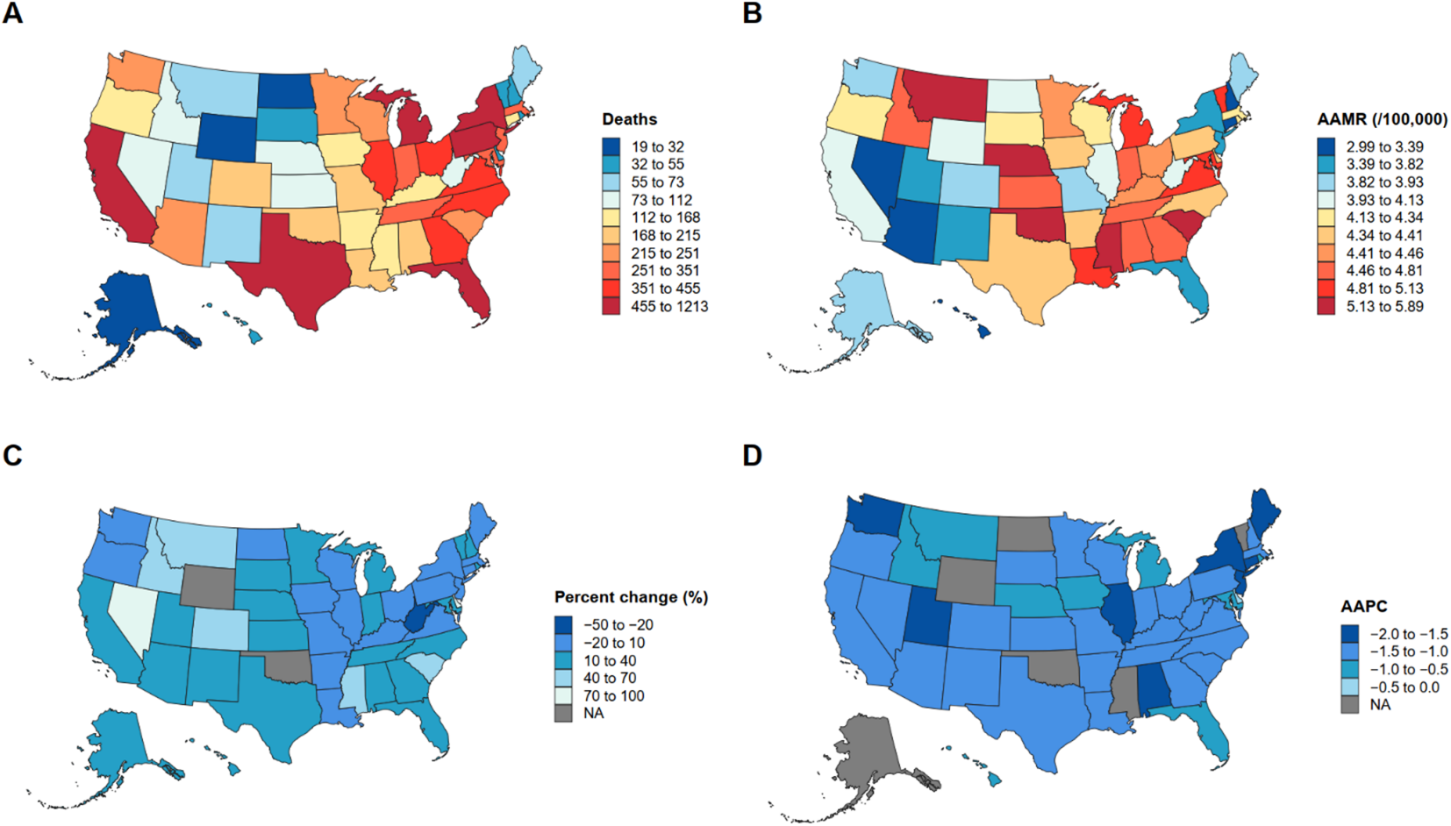
Geographic variation and temporal trends in multiple myeloma–related mortality in the United States, 1999–2023. **(A)** Total number of deaths from multiple myeloma by state. **(B)** Age-adjusted mortality rate (AAMR, per 100,000 population) across states. **(C)** Percent change (%) in mortality between 1999 and 2023. **(D)** Average annual percent change (AAPC) in mortality rates by state.

As shown in **Figure 1A**, in terms of absolute death counts, MM-related deaths were primarily concentrated in highly populated states such as California, Texas, Florida, New York, and Pennsylvania, whereas states with smaller populations—such as Alaska, Wyoming, and Vermont—reported the fewest deaths.

However, after age adjustment, a different spatial pattern emerged (**Figure 1B**). The highest age-adjusted mortality rates (AAMRs) were observed predominantly in the Southeastern and parts of the Midwestern United States, including Mississippi, Alabama, Georgia, South Carolina, Tennessee, Ohio, and Pennsylvania, while relatively lower AAMRs were found in Western and Northern states such as Alaska, Utah, Colorado, Washington, and Minnesota. These findings indicate that, after accounting for age structure, the mortality burden of MM remains higher in the Southeastern and portions of the Central U.S.

**Figure 1C** illustrates the percent change in mortality rates from 1999 to 2023. Most states exhibited a downward trend (ranging from dark to light blue), with significant declines noted in Alaska, Washington, Oregon, California, Texas, and much of the New England region. In contrast, several states such as Oklahoma and West Virginia showed little change or slight increases. Areas shown in gray indicate missing data (NA) due to data suppression.

In terms of average annual percent change (AAPC) (**Figure 1D**), the majority of states demonstrated negative AAPC values, suggesting an overall annual decrease in MM-related mortality. States with the steepest declines included Montana, North Dakota, Washington, and Maine, whereas states such as Oklahoma and West Virginia exhibited smaller decreases, with data missing in a few regions.

Overall, MM mortality in the United States declined between 1999 and 2023, but substantial geographic disparities persisted. Higher age-adjusted mortality rates were concentrated in the Southeastern and Midwestern states, while more pronounced downward trends were observed in the Western and Northern regions, underscoring a clear geographic imbalance in disease burden across the country.

The temporal trends of state-level age-adjusted mortality rates (AAMR) for multiple myeloma from 1999 to 2023 exhibited marked geographic variation (**Supplementary Figure 1**). Overall, most states demonstrated declining AAMR trends; however, the magnitude and segmented patterns of decline varied across states. Some states showed changes in the rate of decline either in the early or more recent periods, reflecting regional differences in disease prevention and control, healthcare resource allocation, and population structure. In general, the majority of states exhibited a significant negative APC from 1999 to 2023 (e.g., Alabama: APC = −1.58, 95% CI: −1.98 to −1.18; Arizona: APC = −1.11, 95% CI: −1.58 to −0.65; California: APC = −1.20, 95% CI: −1.39 to −1.00; Connecticut: APC = −1.63, 95% CI: −2.12 to −1.13; Illinois: APC = −1.60, 95% CI: −1.81 to −1.39; Texas: APC = −1.27, 95% CI: −1.61 to −0.92; Washington: APC = −1.62, 95% CI: −2.04 to −1.19). Meanwhile, several states displayed segmented trends with accelerated declines in recent years. For example, New York exhibited an APC of −0.75 (95% CI: −1.15 to −0.35) from 1999 to 2016, followed by a more rapid decline of −4.64 (95% CI: −6.14 to −3.11) from 2016 to 2023. Ohio showed an APC of −0.70 (95% CI: −1.10 to −0.39) from 1999 to 2018, and −3.79 (95% CI: −6.04 to −1.48) from 2018 to 2023. Kansas had an APC of −0.33 (95% CI: −1.16 to 0.51) from 1999 to 2015, followed by −3.64 (95% CI: −5.96 to −1.27) from 2015 to 2023. In summary, although most states experienced declining AAMR between 1999 and 2023, with some states showing a notably accelerated decline in recent years, there were substantial differences across states in baseline levels, segmented time points, and rates of decrease, reflecting the diversity of regional burden and temporal patterns.

### 3.2 Age-specific analysis

**Figure 2:**
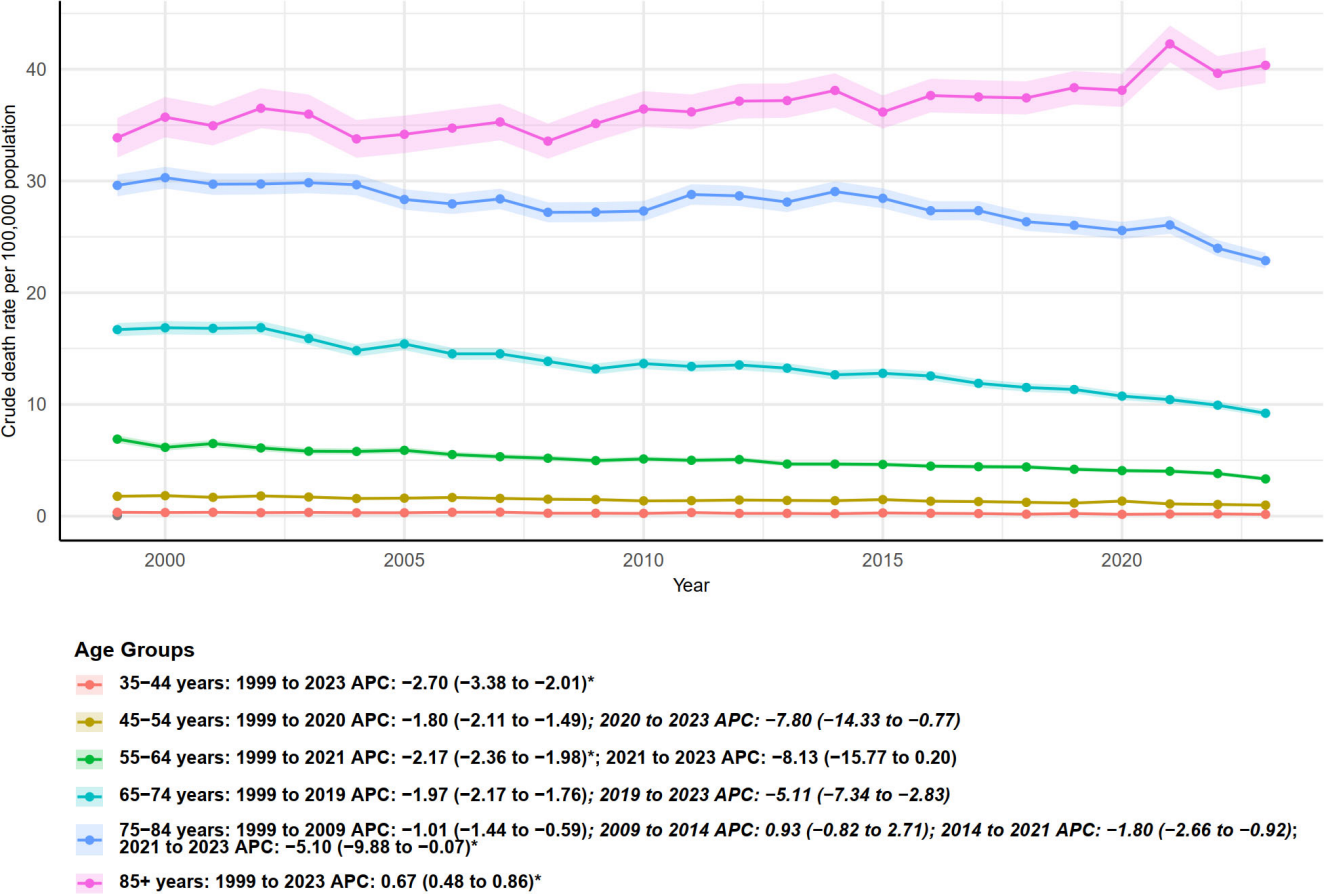
Age-specific trends in crude mortality rates from multiple myeloma in the United States, 1999–2023. Crude death rates (per 100,000 population) are shown for different age groups, with annual percent change (APC) estimates and 95% confidence intervals provided for each period.

**Figure 2** illustrates the trends in crude mortality rates (per 100,000 population) of multiple myeloma from 1999 to 2023, stratified by age group, along with the corresponding annual percent change (APC). A clear age gradient is observed: crude mortality rates increase with age. The 85 years and older group exhibits the highest crude mortality and shows an upward trend, whereas the younger age groups generally demonstrate declining trends, though the onset and pace of these declines vary. Except for the oldest age group (85+), all age groups experienced varying degrees of decrease throughout the study period, with most showing an accelerated decline starting around 2019, 2020, or 2021. However, the continued rise in the 85+ group indicates that the mortality burden of multiple myeloma in the oldest population has not been alleviated, highlighting the need for focused attention in future research and public health strategies.

### 3.3 Regional and urban–rural analysis

**Figure 3:**
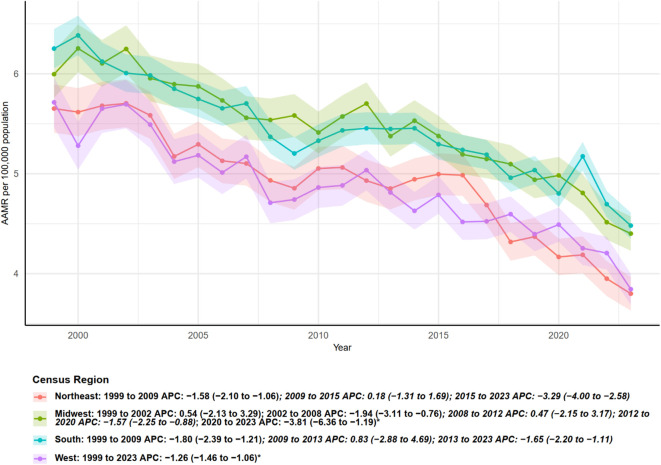
Trends in age-adjusted mortality rates (per 100,000 population) from multiple myeloma with annual percent change (APC) by U.S. census region, 1999–2023.

Age-adjusted mortality rates (AAMR), analyzed according to U.S. Census regions, exhibited regional differences and distinct temporal segment patterns between 1999 and 2023 (**Figure 3**). Northeast: AAMR significantly declined from 1999 to 2009, with an annual percent change (APC) of −1.58 (95% CI: −2.10 to −1.06). The trend stabilized during 2009–2015, followed by an accelerated decrease from 2015 to 2023 (APC = −3.29, 95% CI: −4.00 to −2.58). Overall, the Northeast showed a decreasing trend over the study period, with a pronounced decline after 2015. Midwest: The region exhibited alternating periods of increase and decrease, with the most pronounced and statistically significant decline occurring from 2020 to 2023 (APC = −3.81, 95% CI: −6.36 to −1.19). South: AAMR generally declined, maintaining a significant reduction after 2013 (APC = −1.65, 95% CI: −2.20 to −1.11). West: Throughout the entire study period (1999–2023), AAMR in the West showed a consistent and steady decline, with an overall APC of −1.26 (95% CI: −1.46 to −1.06), which was statistically significant. In summary, all Census regions primarily exhibited decreasing trends; however, the onset, segmented patterns, and recent decline rates varied. The Northeast and West demonstrated long-term stable decreases, whereas the Midwest and South showed segmented fluctuations, with recent years generally reflecting an accelerated downward trend.

**Figure 4:**
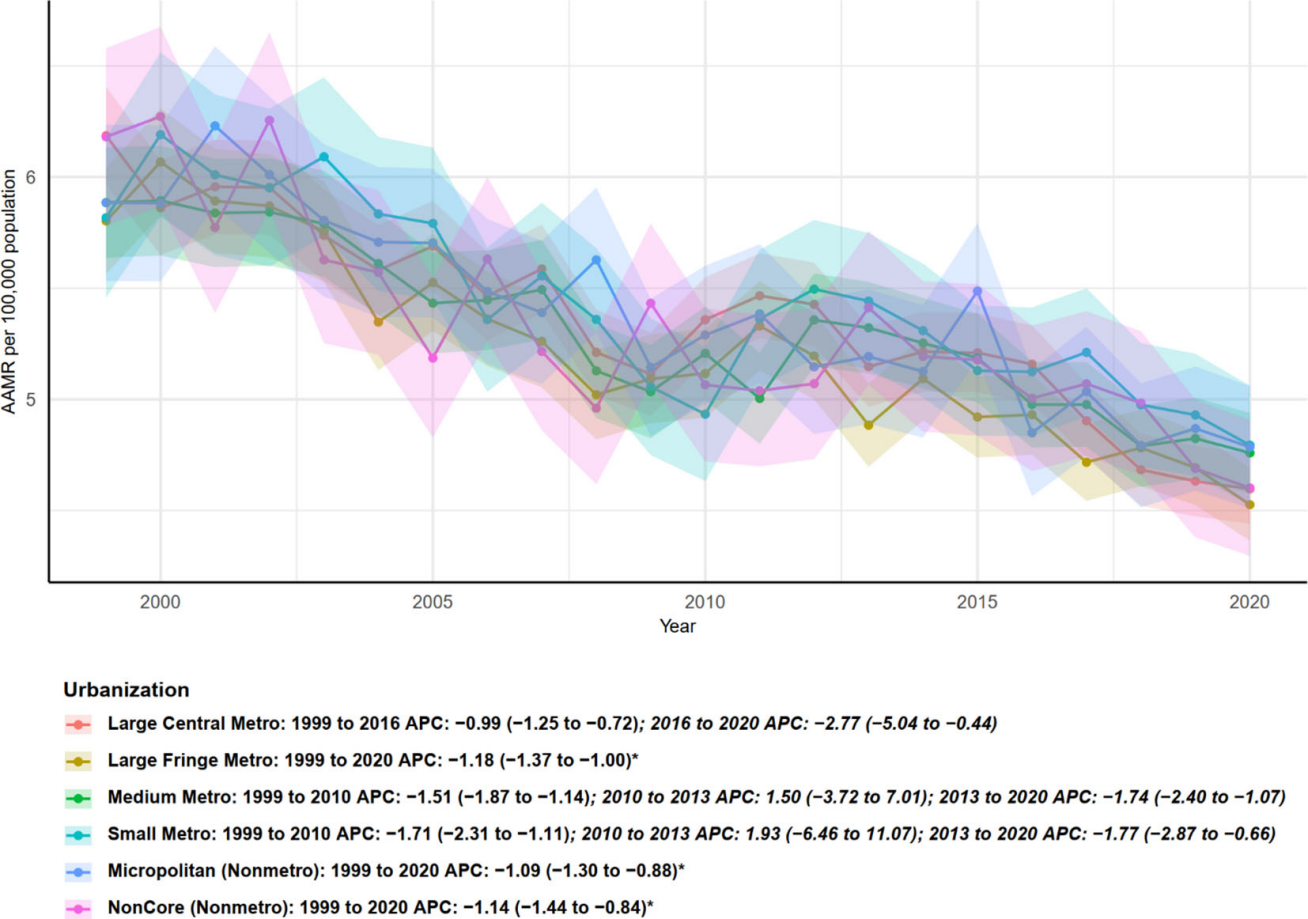
Urban–rural disparities in age-adjusted mortality trends from multiple myeloma by urbanization level, United States, 1999–2020.

Age-adjusted mortality rates (AAMR) stratified by urbanization level—from large central metropolitan areas to noncore nonmetropolitan areas—generally showed a declining trend from 1999 to 2020, although the timing and magnitude of changes varied across urbanization categories (**Figure 4**). Large central metro areas: AAMR declined from 1999 to 2016 (APC = −0.99, 95% CI: −1.25 to −0.72), with an accelerated decrease during 2016–2020 (APC = −2.77, 95% CI: −5.04 to −0.44), indicating a more pronounced decline in recent years. Large fringe metro areas exhibited a stable decline over the entire study period (1999–2020), with an APC of −1.18 (95% CI: −1.37 to −1.00), which was statistically significant. Micropolitan (nonmetro) and noncore (nonmetro) areas, representing the lowest urbanization levels, both showed stable and significant declines from 1999 to 2020, with APCs of −1.09 (95% CI: −1.30 to −0.88) and −1.14 (95% CI: −1.44 to −0.84), respectively. Overall, all urbanization levels exhibited declining AAMR trends, with large central and fringe metro areas showing signs of accelerated decline in recent years. The decline was significant across both urban and rural areas, indicating that multiple myeloma–related mortality decreased regardless of urbanization level, although the timing of segmented changes varied across categories.

### 3.4 Racial/ethnic analysis

**Figure 5:**
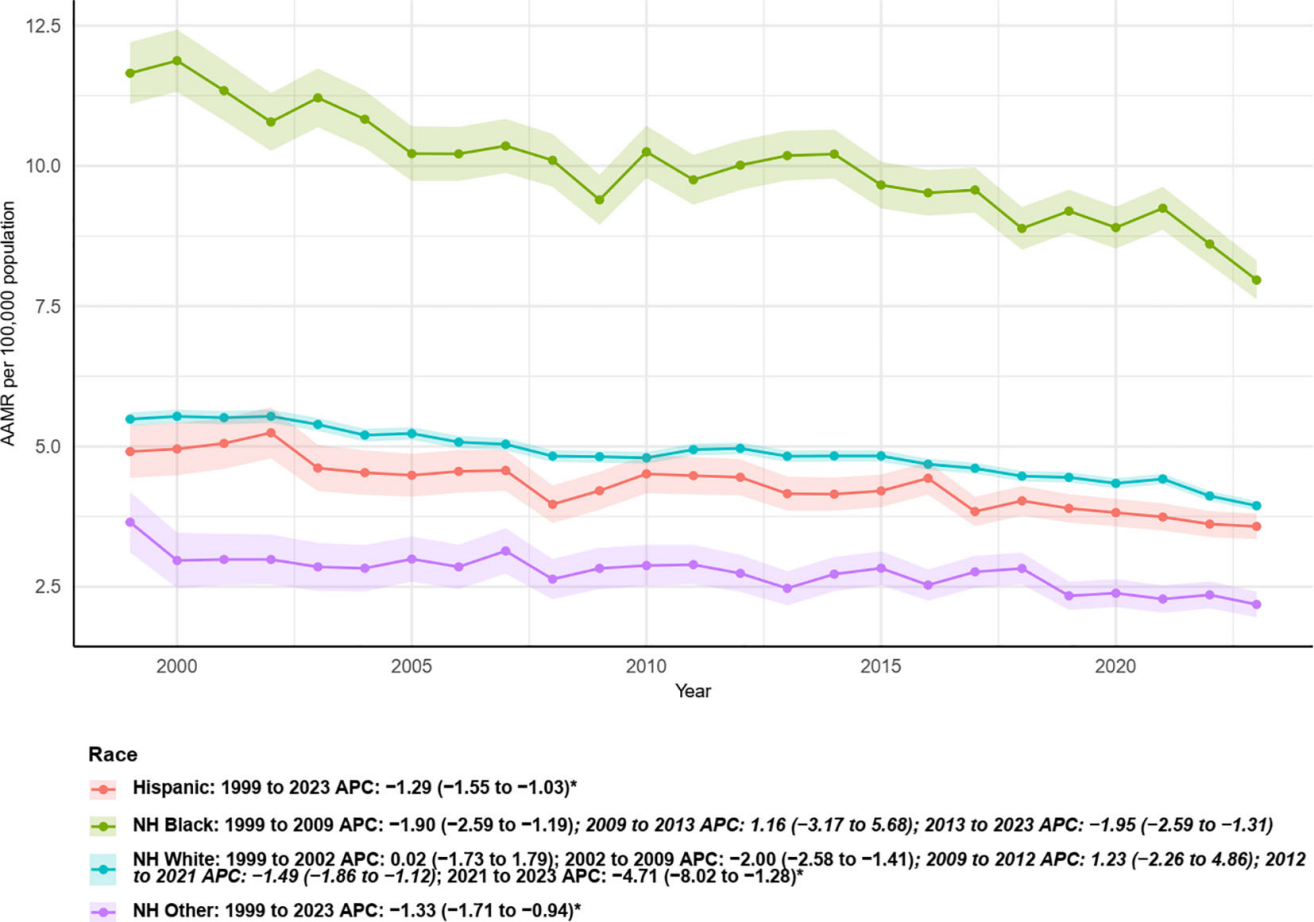
Trends in age-adjusted mortality rates from multiple myeloma with annual percent change (APC) by race, 1999–2023.

**Figure 6:**
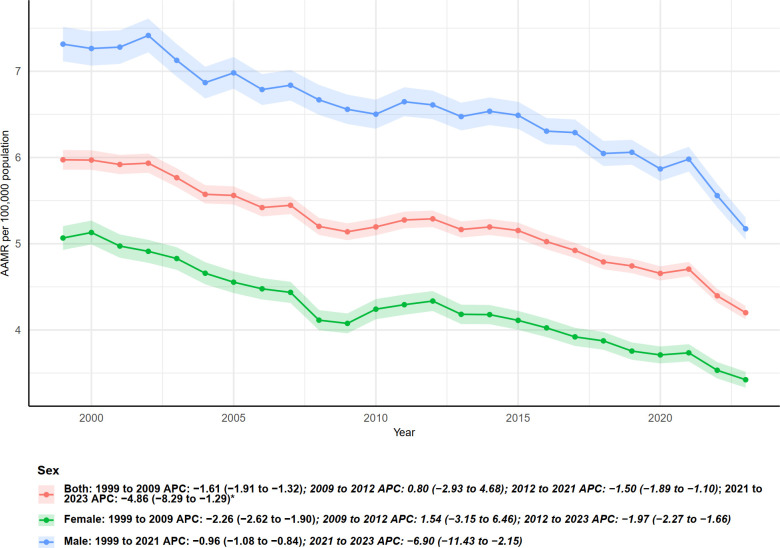
Trends in age-adjusted mortality rates from multiple myeloma with annual percent change (APC) by gender, 1999–2023.

**Figure 5** presents temporal trends in age-adjusted mortality rates (AAMR, per 100,000) and segmented APC estimates for different racial/ethnic groups from 1999 to 2023. Overall, non-Hispanic Black (NH Black) individuals consistently exhibited the highest AAMR among all groups, despite a declining trend beginning in the early study period: 1999–2009 APC = −1.90 (95% CI: −2.59 to −1.19), followed by a brief, non-significant increase during 2009–2013 (APC = 1.16, 95% CI: −3.17 to 5.68). Subsequently, AAMR declined significantly again from 2013 to 2023 (APC = −1.95, 95% CI: −2.59 to −1.31). Non-Hispanic White (NH White) individuals showed an overall stable decline during the study period: 1999–2002 APC = 0.02, 2002–2009 APC = −2.00 (95% CI: −2.58 to −1.41), followed by a non-significant increase from 2009 to 2012, and a subsequent decline from 2012 to 2021 (APC = −1.49, 95% CI: −1.86 to −1.12). The most recent segment, 2021–2023, exhibited a more pronounced decline (APC = −4.71, 95% CI: −8.02 to −1.28). Hispanic individuals also showed a continuous decline over the entire period, with an overall APC of −1.13 from 1999 to 2023. Similarly, the “NH Other” group experienced a steady and significant decrease throughout the study period, with an APC of −1.33 (95% CI: −1.71 to −0.94). Consistently, the lines and confidence intervals in **Figure 6** reflect that although all major racial/ethnic groups have generally declined in recent years, the absolute AAMR among NH Black individuals remains higher than in other groups. Furthermore, the timing and magnitude of declines vary across racial/ethnic groups, highlighting disparities in mortality burden and temporal trends.

### 3.5 Sex-specific analysis

As shown in **Figure 6**, age-adjusted mortality rates (AAMR) and annual percent changes (APC) stratified by sex indicate significant differences in multiple myeloma–related mortality between males and females from 1999 to 2023. Overall, males consistently exhibited higher AAMR than females, although both sexes demonstrated a sustained declining trend. Specifically, for both sexes combined, AAMR declined from 1999 to 2009 (APC = −1.61, 95% CI: −1.91 to −1.32), followed by a slight increase during 2009–2012 (APC = 0.80), then a decline from 2012 to 2021 (APC = −1.50, 95% CI: −1.89 to −1.10), and a more pronounced decrease in 2021–2023 (APC = −4.86, 95% CI: −8.29 to −1.29).

For females, the decline from 1999 to 2009 was more pronounced (APC = −2.26, 95% CI: −2.62 to −1.90), followed by a non-significant increase during 2009–2012 (APC = 1.54, 95% CI: −3.15 to 6.46). From 2012 to 2023, females experienced a continuous decline (APC = −1.97, 95% CI: −2.27 to −1.66).

Males exhibited a smaller decline from 1999 to 2021 (APC = −0.96, 95% CI: −1.08 to −0.84), but showed a significantly accelerated decrease during 2021–2023 (APC = −6.90, 95% CI: −11.43 to −2.15). The shaded areas and lines in **Figure 6** consistently indicate that, despite higher baseline mortality in males, both sexes experienced accelerated declines in the most recent two years (especially 2021–2023), although the significance and magnitude of changes varied across time segments.

## 4 Discussion

This study, based on the 1999–2023 underlying cause of death data from CDC WONDER, systematically delineates the spatiotemporal patterns and stratified disparities in multiple myeloma (MM)–related mortality in the United States. Overall, we observed that, except for the oldest age group (≥85 years) (20), crude mortality rates declined across most age groups during the study period, with several age groups showing accelerated declines in recent years. Concurrently, AAMR exhibited substantial heterogeneity across states and demographic strata—for example, some states experienced accelerated declines in the past decade, whereas others showed slower decreases or early oscillations. These findings are consistent with recent studies based on SEER and CDC WONDER data, which report that although MM incidence has generally increased or remained stable in recent decades, age-adjusted mortality rates have declined overall, yet significant differences persist across racial/ethnic groups and regions (21).

Such geographic and temporal heterogeneity suggests that changes in mortality are unlikely to be driven by a single biological factor; instead, they more plausibly reflect the combined effects of multilevel structural and epidemiologic determinants, for example regional incidence differences, racial and age composition, availability of medical resources and treatment access, socioeconomic status, and variation in data registration and coding practices (19, 22–24). Consequently, when interpreting state-level APC patterns, priority should be given to these mutable ecological and institutional factors rather than to merely restating observed segmented trends. First, interstate differences in incidence and population structure will directly affect AAMR: if a state has experienced a recent increase in MM incidence or a faster rate of population aging, its mortality trajectory may worsen even when treatment capacity is comparable (6, 22). Second, racial composition and socioeconomic inequities — for example the proportions of non-White groups, poverty rates, and educational attainment — can alter mortality outcomes by influencing disease exposure, timeliness of diagnosis, access to care and the comorbidity profile; prior studies show that MM incidence and outcomes are closely linked to race and socioeconomic status, and these factors are highly unevenly distributed across states (13, 25, 26). Third, healthcare resources and treatment accessibility — including the density of oncology and hematology specialists and transplant centers, the diffusion of modern targeted and immune therapies and autologous stem cell transplantation (ASCT), and differences in insurance coverage — determine whether patients receive timely, guideline-concordant therapy and thus shape mortality trends (22, 24, 27). Fourth, public-health shocks and changes in coding or registration practices, such as short-term mortality spikes related to COVID-19 or modifications in cause-of-death attribution, may induce temporary APC fluctuations (28). Finally, data quality issues and small-number variability are more pronounced in less populous states and can exaggerate APC changes near joinpoints. Given these considerations, explanations for state-level variation should move beyond descriptive accounts and toward systematic testing of the measurable covariates outlined above.

Therapeutic advances likely account for a substantial portion of the observed decline in mortality. Since the 2000s, the introduction of proteasome inhibitors, immunomodulatory drugs, monoclonal antibodies, and CAR-T therapies has significantly prolonged both progression-free and overall survival among MM patients, thereby contributing to population-level reductions in mortality. Our observations—for instance, the marked declines in several age groups and states after 2015—temporally coincide with the gradual adoption of these novel treatments, suggesting that treatment accessibility and uptake may have had important effects on population-level mortality trends (29). Despite these therapeutic advances, a significant concern remains the phenomenon of early mortality, defined as death within the first six months following diagnosis. A substantial proportion of patients still succumb to the disease during this critical early period, underscoring that the survival benefits of novel therapies are not universally accessible or effective at presentation (30–32).

However, the benefits of declining mortality have not been equitably distributed across populations. Both this study and prior research have documented pronounced demographic disparities: non-Hispanic Black individuals and males have historically borne higher incidence and mortality burdens (10), while states and urbanization levels exhibit varying temporal patterns and magnitudes of decline (e.g., New York, Ohio, and Kansas showed accelerated declines after certain breakpoints, whereas larger states such as California and Texas, despite high absolute deaths, displayed distinct AAMR trajectories). These disparities likely reflect a confluence of mechanisms, including differences in disease biology (e.g., MGUS prevalence), historical disparities in healthcare access, uneven adoption and benefit of treatments, and regional variations in socioeconomic status and healthcare infrastructure. Recent studies highlighting racial/ethnic and sex-based differences in MM incidence and outcomes further support these explanatory pathways (19, 23, 33–35). CDC WONDER–based analyses indicate that urbanization level exerted differential effects on MM mortality between 1999 and 2020, with patients residing in rural areas experiencing comparatively higher mortality than those in urban settings (36). This disparity likely reflects reduced access to healthcare resources in rural communities, including shortages of specialist physicians (e.g., hematologist–oncologists), limited availability of advanced diagnostic equipment, and fewer treatment facilities such as chemotherapy centers and hematopoietic stem-cell transplantation units. Rural patients often must travel long distances to obtain specialized care, which not only increases financial and logistical burdens but may also contribute to reduced treatment adherence and delays in diagnosis and therapy (34).

Age-stratified results warrant particular attention. Although most young and middle-aged groups (e.g., 35–74 years) showed sustained declines with recent acceleration, the ≥85-year group exhibited a persistent modest increase. This pattern may reflect multiple factors in the oldest patients, including higher comorbidity burdens, poorer functional status, limited tolerance for intensive or novel therapies, and clinical tendencies toward more conservative management. Additionally, underrepresentation of elderly patients in clinical trials may limit the translation of trial-based survival improvements to the oldest age grou These findings underscore the need for targeted assessment and intervention strategies for older MM patients in both public health and clinical practice to avoid “treatment benefit gaps” (29).

We observed a sustained increase in mortality among individuals aged 85 years and older, a trend that can be partly attributed to limited treatment eligibility and inadequate access to medical resources. Older patients are often less likely to receive specialist assessment and complex therapies. On one hand, uneven distribution of oncology and hematology specialists and workforce shortages force elderly patients in remote or resource-poor areas to travel long distances or face prolonged waits for referral, thereby delaying diagnosis and treatment decisions (37). On the other hand, access to advanced diagnostics, such as molecular testing and PET–CT, and to complex treatment facilities, including chemotherapy centers or transplant units experienced in the care of older adults, is uneven across regions and institutions; as a result, some older patients with potentially treatable disease cannot receive optimal or potentially beneficial therapies (38). Consequently, the United States and other regions face a risk of inadequate oncology and hematology workforce coverage for the elderly and systemic barriers to transplant and high-intensity therapies. These structural access issues can produce an age-based “exclusion” effect that contributes to rising mortality in the very-old age grou When interpreting the increase in mortality among those aged 85 and older, it is therefore important to consider “healthcare resource and specialist accessibility” as a key structural driver and to emphasize the inclusion of variables such as oncology and transplant center density, referral distance, and insurance coverage in national or state-level ecological analyses to test this mechanism.

Comorbidity burden provides another critical pathway explaining the rising mortality in the oldest age grou Compared with younger patients, individuals aged 85 and older carry a higher burden of multiple chronic conditions — cardiovascular disease, diabetes, chronic respiratory disease and others — and are more likely to be excluded from standard cancer treatment pathways because of these comorbidities (39, 40). The literature shows that comorbidity substantially worsens cancer outcomes and, in patients with localized or regional disease, often reduces overall survival by increasing non-cancer competing causes of death (41, 42). Thus, in interpreting the mortality increase in this age stratum, it is essential to disentangle contributions from cancer-specific deaths and deaths related to non-cancer comorbidities, and to consider treatment modifications induced by comorbidity (dose reductions or treatment discontinuation) as mediating variables. Incorporating comorbidity indices such as the Charlson Comorbidity Index into survival or temporal trend models can help quantify the contribution of comorbidity to APC changes. Likewise, frailty is an independent predictor of outcomes in older oncology patients: it is associated with higher treatment-related toxicity and complication rates and with worse overall survival (43). Numerous studies have shown that baseline frailty or prefrailty in older patients receiving chemotherapy or other systemic therapies significantly increases the risk of severe adverse events, reduces quality of life, and elevates short- to mid-term mortality (44). Consequently, clinicians often adopt conservative strategies based on functional status or frailty assessments, including dose reductions or the avoidance of aggressive therapy, to minimize toxicity. Although such caution may reduce acute harms, it can also lead to potentially avoidable cancer deaths or the loss of curative opportunities at the population level, and thus contribute to the observed rise in mortality among those aged 85 and older (45). For these reasons, explanations of increasing mortality in the elderly should incorporate “treatment selection and under-treatment driven by frailty” into the causal chain. We therefore recommend routine inclusion of simple frailty screening tools, such as clinical frailty scales or the G8, in epidemiologic studies and clinical practice to better distinguish deaths resulting from treatment avoidance due to frailty from deaths attributable to end-stage disease (46).

Patients with multiple myeloma (MM) are inherently immunocompromised, rendering them more susceptible to infections, which remain among the leading causes of mortality in this population. Sepsis, in particular, represents a major contributor to infection-related deaths. A nationwide analysis of sepsis-associated mortality among MM patients from 1999 to 2020 reported a declining overall trend; however, the persistent burden of this complication warrants ongoing clinical vigilance (47). In addition, respiratory infections such as pneumonia, influenza, and COVID-19 constitute important causes of death in MM patients. An analysis of national mortality data from 1999 to 2022 revealed dynamic temporal changes in mortality attributable to these infections, with markedly elevated risks and disease severity observed among MM patients during the COVID-19 pandemic period (28, 48). Similar limitations are common to population-based death certificate studies and have been previously discussed in the literature (12).

This study leverages nationally representative CDC WONDER data over a long period (1999–2023) and examines multiple stratifications, including sex, age, race/ethnicity, state, and urbanization level. This allows a systematic assessment of MM mortality patterns and potential inequities at the national level. Such population-level, long-term ecological analyses provide baseline evidence for public health decision-making and help identify high-burden populations and regions that warrant prioritization for intervention (21).

Based on our findings, future research and policy directions should include: first, cohort or registry studies linking diagnosis, treatment, and outcomes to elucidate how treatment uptake, effectiveness, and social determinants drive population-level mortality changes; second, the design and evaluation of interventions to improve access, optimize care, and facilitate early diagnosis and treatment for high-burden populations, such as non-Hispanic Black individuals, older patients, and states with slower declines; and third, enhancing the representation of vulnerable populations (e.g., elderly, racial/ethnic minorities) in clinical trials to ensure equitable benefits from novel therapies. Overall, although population-level MM mortality has declined, research and policy efforts must prioritize “equitable benefit” to ensure that mortality improvements do not create or exacerbate health disparities across populations (29).

## 5 Limitation

Several methodological limitations should be noted. First, CDC WONDER data are derived from death certificates, in which the underlying cause of death is recorded by certifiers and may be subject to diagnostic and coding inaccuracies, potentially leading to misclassification or systematic bias. Additionally, WONDER suppresses data for cells with low counts, which may affect the precision of estimates for small subpopulations or certain states. Second, mortality databases do not provide individual-level information on treatment exposure, pathological staging, comorbidities, or socioeconomic status, limiting our ability to attribute regional or population-level mortality differences directly to treatment accessibility or social determinants. Third, although our study spans 1999–2023 and captures long-term trends, short-term jumps in recent years (e.g., the rapid decline observed from 2020–2023) should be interpreted cautiously, as they may reflect reporting delays, changes in clinical practice, or statistical segmentation methods.

## 6 Conclusion

Using CDC WONDER national data, we analyzed U.S. multiple myeloma (MM)–related mortality from 1999–2023. Although national age-adjusted mortality rates (AAMRs) declined overall, substantial disparities persisted by age, sex, race/ethnicity, and geography: mortality increased among those aged ≥85; males had higher AAMRs than females, though declines in both accelerated recently; non-Hispanic Black individuals consistently had the highest AAMR. Geographically, the Southeast and Midwest bore higher burdens while the West and North showed larger declines; New York, Ohio, and Kansas exhibited accelerated reductions, whereas Oklahoma and West Virginia showed little change. All urbanization levels experienced declines, with large metropolitan areas seeing faster recent decreases. The findings underscore the need for targeted public-health strategies to ensure equitable access to effective therapies and for linking clinical and social determinants to mortality trends to guide interventions and resource allocation.

## Data Availability

The original contributions presented in the study are included in the article/**Supplementary Material**. Further inquiries can be directed to the corresponding author.

## Supplementary material

Supplementary Figure 1 Trends in age-adjusted mortality rates from multiple myeloma with annual percent change (APC) by state, 1999–2023.
