# Supplementary material for: Trends in mortality rates associated with multiple myeloma in the United States, 1999–2023
Source: Front Oncol. 2025 Dec 12;15:1735565. doi: 10.3389/fonc.2025.1735565 (PMC12740889; doi:10.3389/fonc.2025.1735565)
Supplement: Supplementary Figure 1 — Trends in age-adjusted mortality rates from multiple myeloma with annual percent change (APC) by state, 1999–2023. [file Image1.pdf]

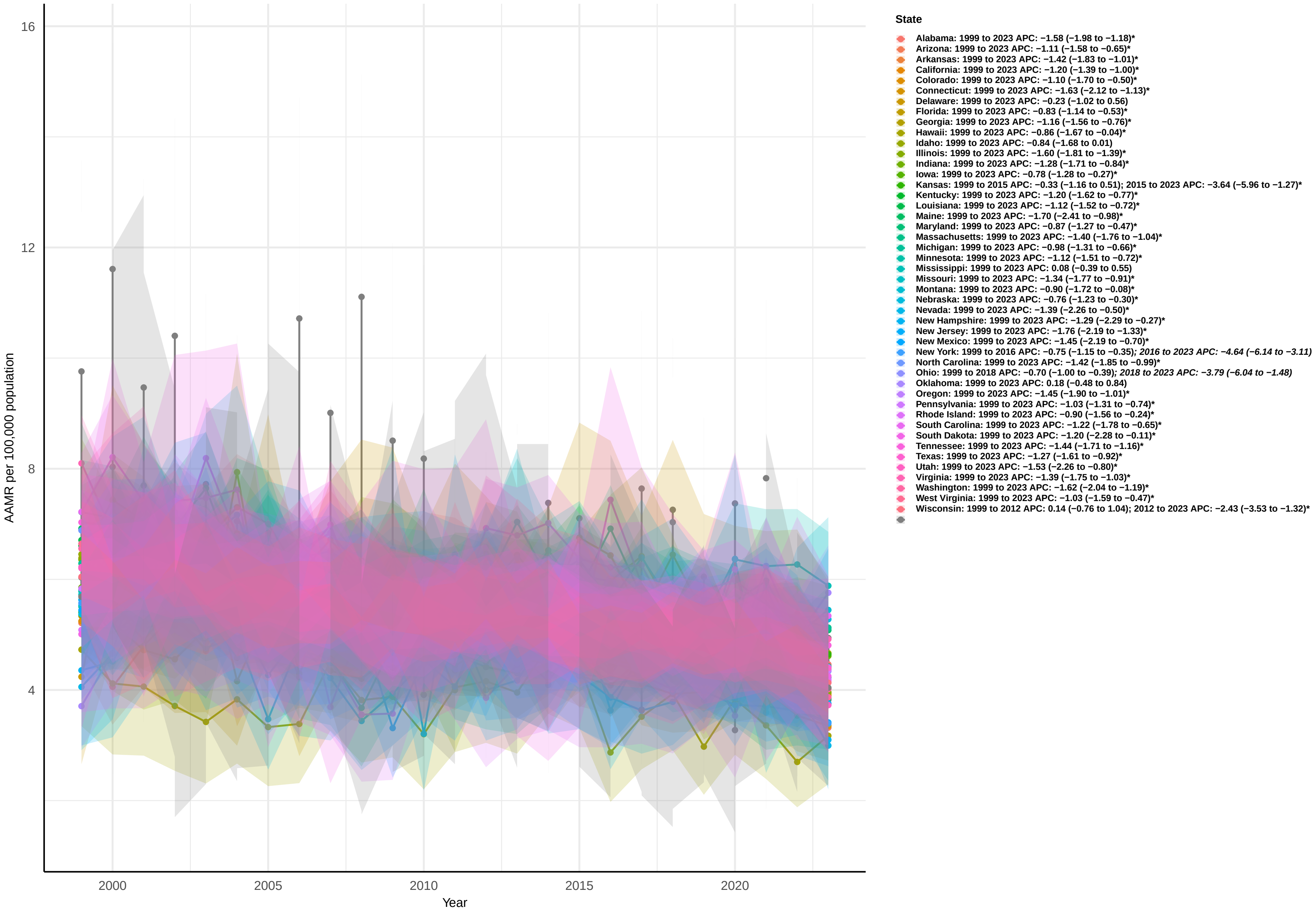
16
12
8
4
AAMR per 100,000 population
2000
2005
2010
2015
2020
Year
State
Alabama: 1999 to 2023 APC: −1.58 (−1.98 to −1.18)*
Arizona: 1999 to 2023 APC: −1.11 (−1.58 to −0.65)*
Arkansas: 1999 to 2023 APC: −1.42 (−1.83 to −1.01)*
California: 1999 to 2023 APC: −1.20 (−1.39 to −1.00)*
Colorado: 1999 to 2023 APC: −1.10 (−1.70 to −0.50)*
Connecticut: 1999 to 2023 APC: −1.63 (−2.12 to −1.13)*
Delaware: 1999 to 2023 APC: −0.23 (−1.02 to 0.56)
Florida: 1999 to 2023 APC: −0.83 (−1.14 to −0.53)*
Georgia: 1999 to 2023 APC: −1.16 (−1.56 to −0.76)*
Hawaii: 1999 to 2023 APC: −0.86 (−1.67 to −0.04)*
Idaho: 1999 to 2023 APC: −0.84 (−1.68 to 0.01)
Illinois: 1999 to 2023 APC: −1.60 (−1.81 to −1.39)*
Indiana: 1999 to 2023 APC: −1.28 (−1.71 to −0.84)*
Iowa: 1999 to 2023 APC: −0.78 (−1.28 to −0.27)*
Kansas: 1999 to 2015 APC: −0.33 (−1.16 to 0.51); 2015 to 2023 APC: −3.64 (−5.96 to −1.27)*
Kentucky: 1999 to 2023 APC: −1.20 (−1.62 to −0.77)*
Louisiana: 1999 to 2023 APC: −1.12 (−1.52 to −0.72)*
Maine: 1999 to 2023 APC: −1.70 (−2.41 to −0.98)*
Maryland: 1999 to 2023 APC: −0.87 (−1.27 to −0.47)*
Massachusetts: 1999 to 2023 APC: −1.40 (−1.76 to −1.04)*
Michigan: 1999 to 2023 APC: −0.98 (−1.31 to −0.66)*
Minnesota: 1999 to 2023 APC: −1.12 (−1.51 to −0.72)*
Mississippi: 1999 to 2023 APC: 0.08 (−0.39 to 0.55)
Missouri: 1999 to 2023 APC: −1.34 (−1.77 to −0.91)*
Montana: 1999 to 2023 APC: −0.90 (−1.72 to −0.08)*
Nebraska: 1999 to 2023 APC: −0.76 (−1.23 to −0.30)*
Nevada: 1999 to 2023 APC: −1.39 (−2.26 to −0.50)*
New Hampshire: 1999 to 2023 APC: −1.29 (−2.29 to −0.27)*
New Jersey: 1999 to 2023 APC: −1.76 (−2.19 to −1.33)*
New Mexico: 1999 to 2023 APC: −1.45 (−2.19 to −0.70)*
New York: 1999 to 2016 APC: −0.75 (−1.15 to −0.35); 2016 to 2023 APC: −4.64 (−6.14 to −3.11)
North Carolina: 1999 to 2023 APC: −1.42 (−1.85 to −0.99)*
Ohio: 1999 to 2018 APC: −0.70 (−1.00 to −0.39); 2018 to 2023 APC: −3.79 (−6.04 to −1.48)
Oklahoma: 1999 to 2023 APC: 0.18 (−0.48 to 0.84)
Oregon: 1999 to 2023 APC: −1.45 (−1.90 to −1.01)*
Pennsylvania: 1999 to 2023 APC: −1.03 (−1.31 to −0.74)*
Rhode Island: 1999 to 2023 APC: −0.90 (−1.56 to −0.24)*
South Carolina: 1999 to 2023 APC: −1.22 (−1.78 to −0.65)*
South Dakota: 1999 to 2023 APC: −1.20 (−2.28 to −0.11)*
Tennessee: 1999 to 2023 APC: −1.44 (−1.71 to −1.16)*
Texas: 1999 to 2023 APC: −1.27 (−1.61 to −0.92)*
Utah: 1999 to 2023 APC: −1.53 (−2.26 to −0.80)*
Virginia: 1999 to 2023 APC: −1.39 (−1.75 to −1.03)*
Washington: 1999 to 2023 APC: −1.62 (−2.04 to −1.19)*
West Virginia: 1999 to 2023 APC: −1.03 (−1.59 to −0.47)*
Wisconsin: 1999 to 2012 APC: 0.14 (−0.76 to 1.04); 2012 to 2023 APC: −2.43 (−3.53 to −1.32)*
