# Supplementary material for: Trends in mortality rates associated with multiple myeloma in the United States, 1999–2023
Source: Front Oncol. 2025 Dec 12;15:1735565. doi: 10.3389/fonc.2025.1735565 (PMC12740889; doi:10.3389/fonc.2025.1735565)
Supplement: Supplementary file 2 [file Table1.docx]

| Measure | deaths.all |
| --- | --- |
| 35-44 years | 2822 |
| 45-54 years | 15170 |
| 55-64 years | 43685 |
| 85+ years | 50948 |
| 65-74 years | 79455 |
| 75-84 years | 96624 |
| 25-34 years |  |
| Northeast | 52880 |
| West | 57805 |
| Midwest | 67394 |
| South | 110867 |
| NH Other | 6913 |
| Hispanic | 18681 |
| NH Black | 50718 |
| NH White | 212000 |
| Female | 133545 |
| Male | 155401 |
| Both | 288946 |
| Vermont | 615 |
| North Dakota | 728 |
| District of Columbia | 735 |
| South Dakota | 897 |
| Rhode Island | 983 |
| Delaware | 985 |
| Hawaii | 1002 |
| Montana | 1062 |
| New Hampshire | 1247 |
| Idaho | 1407 |
| Maine | 1453 |
| New Mexico | 1722 |
| Nebraska | 1741 |
| Utah | 1797 |
| Nevada | 1926 |
| West Virginia | 2073 |
| Kansas | 2856 |
| Arkansas | 2915 |
| Mississippi | 3086 |
| Iowa | 3300 |
| Connecticut | 3311 |
| Oklahoma | 3568 |
| Colorado | 3887 |
| Oregon | 3967 |
| Kentucky | 4243 |
| Louisiana | 4577 |
| Minnesota | 5178 |
| Alabama | 5216 |
| South Carolina | 5369 |
| Arizona | 5388 |
| Wisconsin | 5682 |
| Missouri | 6014 |
| Washington | 6021 |
| Maryland | 6059 |
| Indiana | 6234 |
| Massachusetts | 6241 |
| Tennessee | 6737 |
| Virginia | 7850 |
| New Jersey | 8191 |
| Georgia | 8373 |
| North Carolina | 9824 |
| Michigan | 10727 |
| Illinois | 11712 |
| Ohio | 12325 |
| Pennsylvania | 13889 |
| New York | 16950 |
| Texas | 18983 |
| Florida | 20274 |
| California | 28771 |
| Alaska |  |
| Wyoming |  |
| NonCore (Nonmetro) | 19729 |
| Micropolitan (Nonmetro) | 25167 |
| Small Metro | 25311 |
| Medium Metro | 53539 |
| Large Fringe Metro | 59013 |
| Large Central Metro | 69658 |

Supplementary table 1. The total number of deaths among patients aged 25 with multiple myeloma in the United States from 1999 to 2023
